# Supplementary material for: The molecular basis of JAK/STAT inhibition by SOCS1
Source: Nat Commun. 2018 Apr 19;9:1558. doi: 10.1038/s41467-018-04013-1 (PMC5908791; doi:10.1038/s41467-018-04013-1)
Supplement: Supplementary file 1 — Supplementary Information [file 41467_2018_4013_MOESM1_ESM.pdf]

Supplementary Material

The molecular basis of JAK/STAT inhibition by SOCS1

Nicholas P.D. Liau[1,2], Artem Laktyushin[1,2], Isabelle S. Lucet[1,2], James M. Murphy[1,2], Shenggen Yao[2], Eden Whitlock[1,2], Kimberley Callaghan[1,2], Nicos A. Nicola[1,2], Nadia J. Kershaw[1,2]* and Jeffrey J. Babon[1,2]*

[1]*Walter and Eliza Hall Institute, 1G Royal Parade, Parkville, 3052, VIC, Australia*

[2]*The University of Melbourne, Royal Parade, Parkville, 3050, VIC, Australia*

*Correspondence: Email: babon@wehi.edu.au, kershaw@wehi.edu.au, Tel +61 3 93452960; Fax +61 3 93470852

*These authors contributed equally

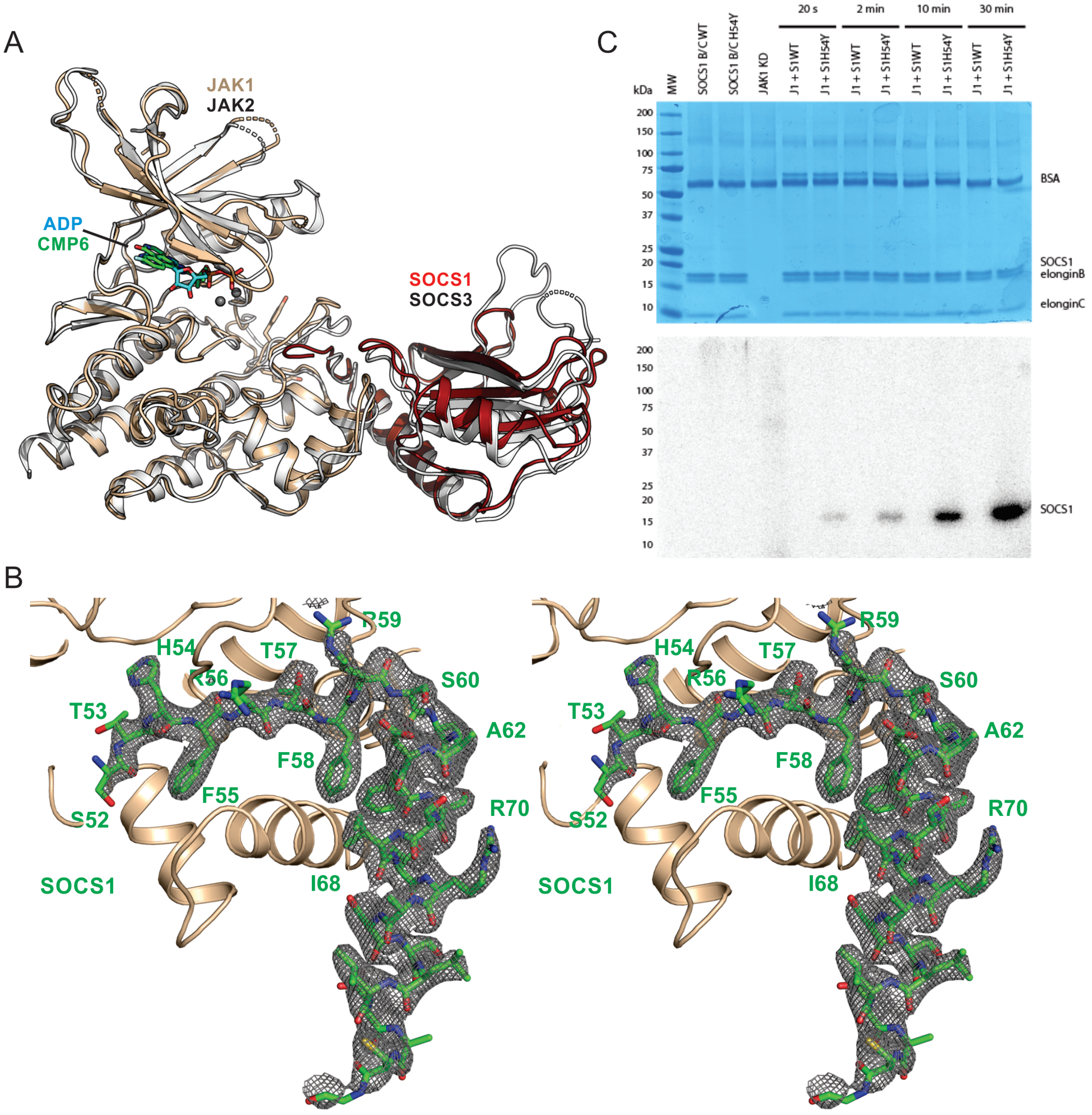

Supplemental Figure 1. The structure of SOCS1/JAK1. (A) Overlaid structures of the JAK1-SOCS1 complex (beige and red) and the JAK2-SOCS3-gp130 complex (white) indicate overall structural similarity. (B) Stereo representation of 2Fo - Fc electron density map of the KIR region of SOCS1 (green) contoured at 1.5σ. (C) SOCS1 H54Y is efficiently phosphorylated by JAK1. ^{32}P-γ- ATP was incubated with 1 μM JAK1 and 5 μM SOCS1 for the indicated times followed by SDS-PAGE (upper) and phosphorimaging (lower). As shown H54Y SOCS1 is robustly phosphorylated whereas WT SOCS1 is not.

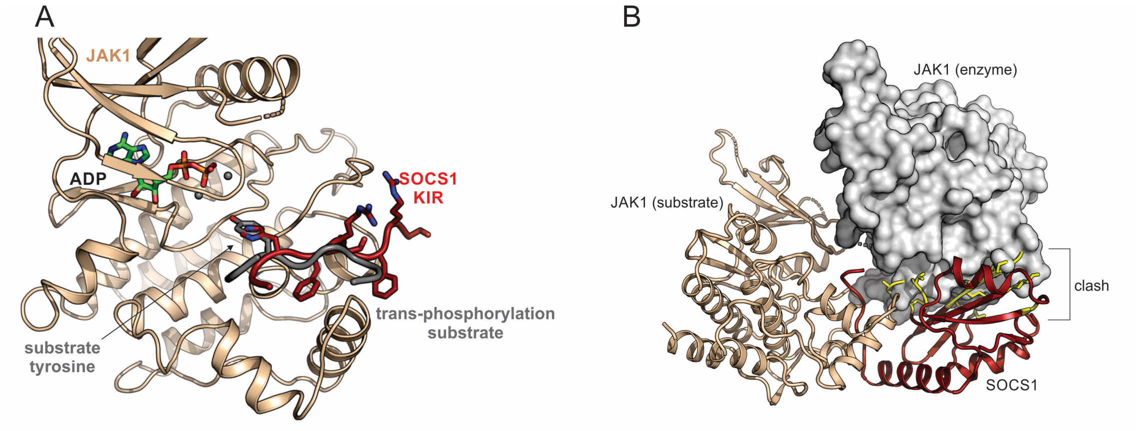

Supplementary Figure 2. SOCS1 hinders JAK1 transphosphorylation.

(A) SOCS1 blocks transactivation of a second JAK molecule by blocking the activation loop binding site. The SOCS1/JAK1 structure is shown overlaid with a model of the activation loop from a transphosphorylation substrate. Based on the IGF1 receptor kinase in its transphosphorylation-competent dimeric state (PDB ID: 3D94). (B) The structure of JAK1 (beige) in complex with SOCS1 (red) is shown. In order for the JAK1 activation loop Tyr1034 to be transphosphorylated Tyr1034 (yellow) a second JAK molecule (acting as the enzyme) must interact with the complex. Here we have modelled in the second JAK molecule (grey surface) based on the structure of the IGF1 receptor tyrosine kinase in its trans-phosphorylation state (PDB ID: 3D94 [28]) which can be seen to clash with the SOCS1 SH2 domain.

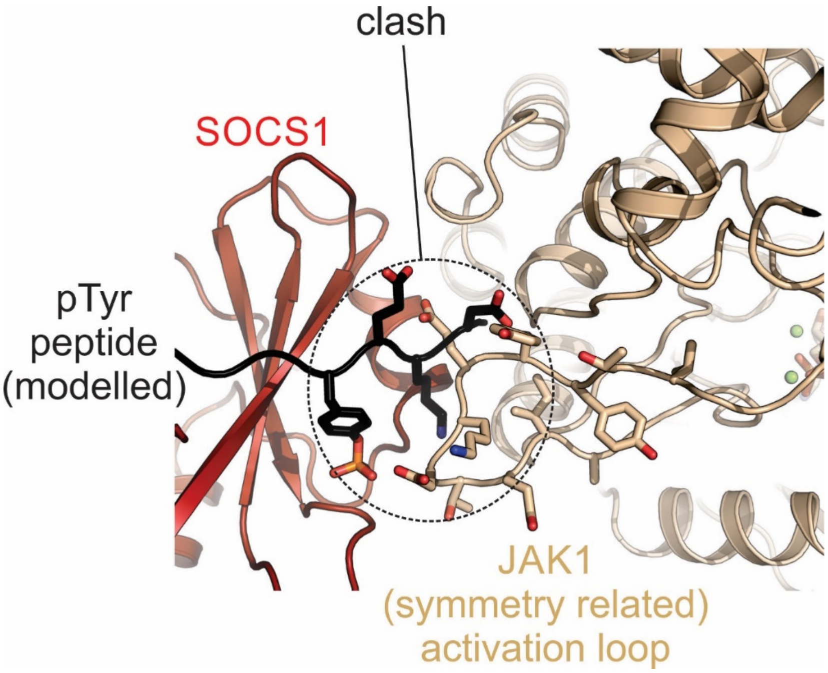

Supplementary Figure 3. Crystal packing prevents SOCS1 binding phosphopeptide. SOCS1 (Red) with a model of phosphopeptide binding canonical SH2 domain binding site (Black), based on SOCS3/gp130 peptide structure (PDB: 4GL9). A symmetry related JAK1 molecule is shown in beige. The neighbouring JAK activation loop partially occludes the peptide binding site by blocking residues N-terminal to the phosphotyrosine.

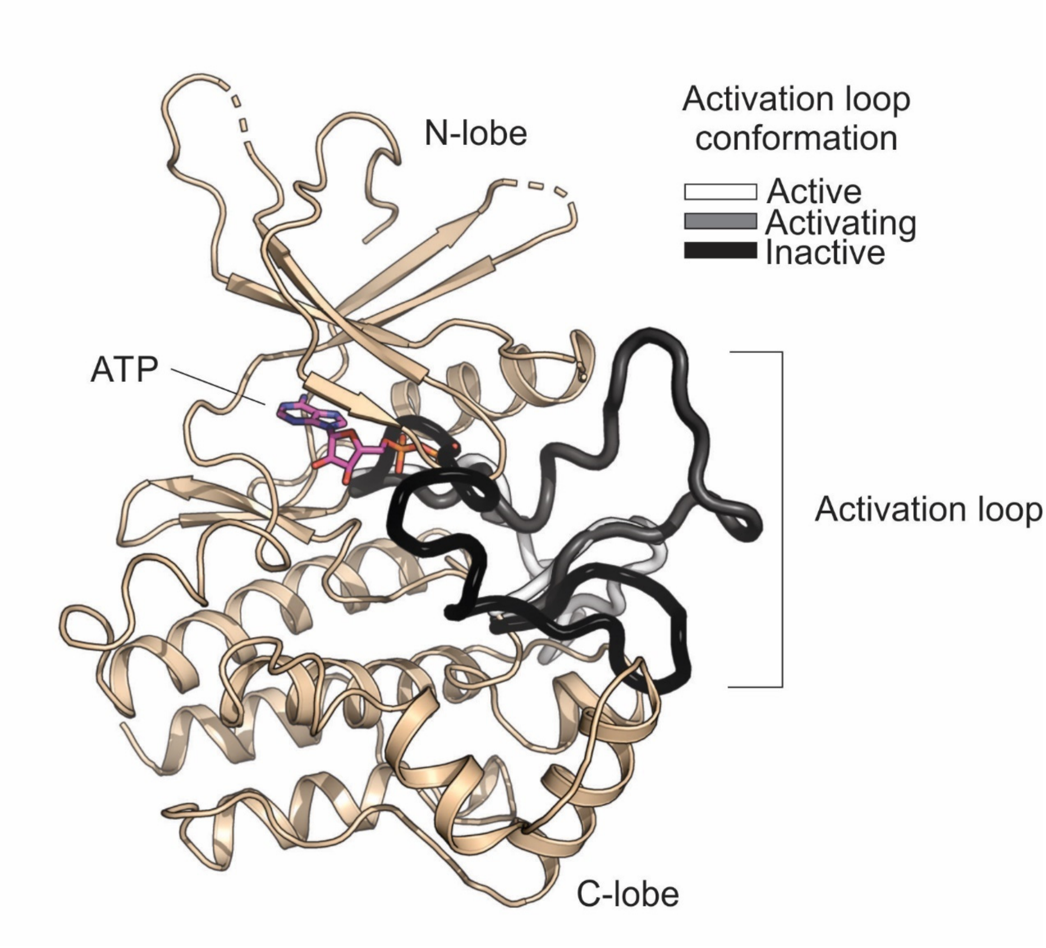

Supplementary Figure 4. The activation loop is a mobile element. The activation loop is shown modelled in its inactive (black, derived from the insulin receptor kinase structure: PDB 1IRK) and active (white, this manuscript PDB: 6C7Y) forms. In addition it is shown modelled in its "activating" conformation in which it is configured to be phosphorylated by a second kinase molecule (grey, derived from the IGF1 receptor kinase structure, PDB: 3D94 [28]).

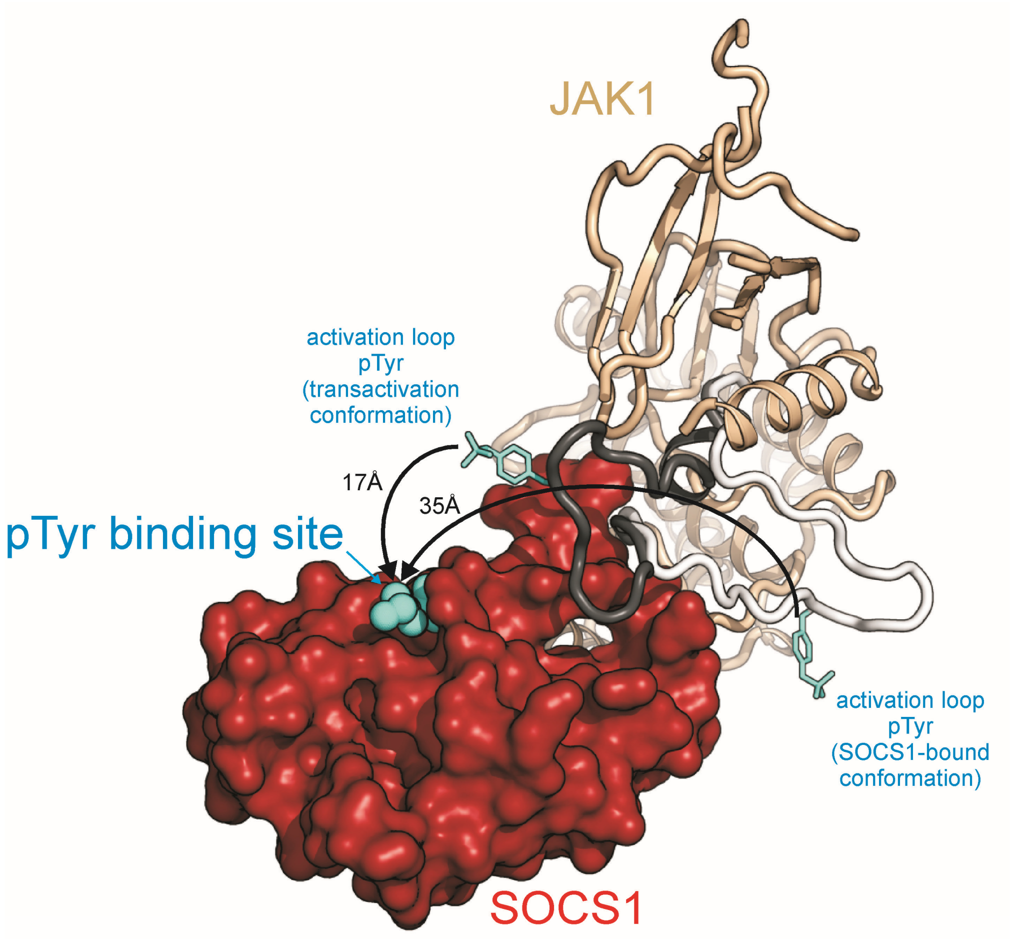

Supplementary Figure 5. JAK1 activation loop displacement. The JAK1 activation loop would require a large displacement (ca. 35Å) in order to bind the SH2 domain of the bound SOCS1 molecule. However, if the activation loop were able to adopt its "activating conformation" [28] then the displacement required is 17Å.

Supplementary Table 1: Peptide sequences used for isothermal titration calorimetry

Peptide	Sequence
FLAG	DYKDDDDK
STAT5b	RRAKAADGYVKPQIKQVV
JAK1 ALP no pY	AIETDKEYYTVKDDRD
JAK1 ALP 1pY	AIETDKE[pY]YTVKDDRD
JAK1 ALP 2pY	AIETDKE[pY][pY]TVKDDLD
JAK1 ALP 1pY short	DKE[pY]YTVKDDRD
JAK2 ALP 2pY	VLPQDKE[pY][pY]KVKEPGE
JAK3 ALP 2pY	LLPLDKD[pY][pY]VVREPGQ
TYK2 ALP 2pY	VPEGHE[pY][pY]RVREDGD
JAK1 ALP ala pY-7	**E**IETDKE[pY]YTVKDDRD
JAK1 ALP ala pY-6	A**A**ETDKE[pY]YTVKDDRD
JAK1 ALP ala pY-5	AI**A**TDKE[pY]YTVKDDRD
JAK1 ALP ala pY-4	AIE**A**DKE[pY]YTVKDDRD
JAK1 ALP ala pY-3	AIET**A**KE[pY]YTVKDDRD
JAK1 ALP ala pY-2	AIETD**A**E[pY]YTVKDDRD
JAK1 ALP ala pY-1	AIETDK**A**[pY]YTVKDDRD
JAK1 ALP ala pY+1	AIETDKE[pY]**A**TVKDDRD
JAK1 ALP ala pY+2	AIETDKE[pY]Y**A**VKDDRD
JAK1 ALP ala pY+3	AIETDKE[pY]YT**A**KDDRD
JAK1 ALP ala pY+4	AIETDKE[pY]YTV**A**DDRD
JAK1 ALP ala pY+5	AIETDKE[pY]YTVK**A**DRD
JAK1 ALP ala pY+6	AIETDKE[pY]YTVKD**A**RD
JAK1 ALP ala pY+7	AIETDKE[pY]YTVKDD**A**D
JAK1 ALP ala pY+8	AIETDKE[pY]YTVKDDR**A**
IFNGR1.1	ICF[pY]IKKINPLK
IFNGR1.2	ESK[pY]VSLITSYQ
IFNGR1.3	ITS[pY]QPFSLEKE
IFNGR1.4	LNS[pY]HSRNCSES
IFNGR1.5	SFG[pY]DKPHVLVD
IFNGR1.6	LIG[pY]RPTEDSKE
IFNGR2.1	VLK[pY]RGLIKYWF
IFNGR2.2	LIK[pY]WFHTPPSI
IFNGR2.3	IEE[pY]LKDPTQPI
γc.1	VTE[pY]HGNFSAWS
γc.2	QPD[pY]SERLCLVS
γc.3	HSP[pY]WAPPCYTL
γc.4	PPC[pY]TLKPET
IL-2RB.1	TNQG[pY]FFFHLPDA
IL-2RB.2	CQV[pY]FTYDPYSE
IL-2RB.3	YFT[pY]DPYSEEDP
IL-2RB.4	YDP[pY]SEEDPDEG
IL-2RB.5	DDA[pY]CTFPSRDD
IL-2RB.6	NTDA[pY]LSLQELQG

IFNAR1.1	RCIN[pY]VFFPSL
IFNAR1.2	SIDE[pY]FSEQPL
IFNAR1.3	DHKK[pY]SSQTSQ
IFNAR1.4	DSGN[pY]SNEDES
IFNAR2.1	KWIG[pY]ICLRNS
IFNAR2.2	VEVI[pY]INRKKK
IFNAR2.3	KVWD[pY]NYDDES
IFNAR2.4	WDYN[pY]DDESDS
IFNAR2.5	SGGG[pY]TMHGLT
IFNAR2.6	PEED[pY]SSTEGS
IFNAR2.7	DLGDG[pY]IMR

Supplementary Table 2: ITC results of activation loop peptide alanine scan

**Peptide**	**K_d ± SEM (nM)**	**ΔH ± SEM (kcal/mol)**
WT	131.5 ± 66.5	-6.5 ± 0.5
pY-7	115.8 ± 0.3	-3.5 ± 2.2
pY-6	140.5 ±13.9	-6.2 ± 0.3
pY-5	371.0 ± 5.0	-3.7 ± 0.7
pY-4	188.9 ±122.7	-6.9 ± 0.1
pY-3	252.8 ± 21.9	-6.6 ± 2.2
pY-2	383.2 ±10.2	-6.7 ± 0.2
pY-1	390.8 ± 45.9	-6.2 ± 0.7
pY+1	159.3 ± 121.6	-4.4 ± 0.6
pY+2	266.3 ± 75.1	-6.5 ± 0.9
pY+3	1397 ± 297.2	-2.1 ± 1.5
pY+4	213.6 ± 27.4	-9.3 ± 0.6
pY+5	1116 ± 391.8	-5.3 ± 0.3
pY+6	488.2 ± 231.2	-7.2 ± 1.2
pY+7	322.6 ± 129.9	-8.2 ± 0.3
pY+8	232.0 ±55.9	-6.0 ± 0.3
